# Mapping the human parafoveal vascular network to understand flow variability in capillaries

**DOI:** 10.1371/journal.pone.0292962

**Published:** 2023-10-13

**Authors:** Srividya Neriyanuri, Phillip Bedggood, R. C. Andrew Symons, Andrew Metha

**Affiliations:** 1 Department of Optometry and Vision Sciences, The University of Melbourne, Victoria, Australia; 2 Department of Surgery, The University of Melbourne, Victoria, Australia; 3 Centre for Eye Research Australia, Victoria, Australia; 4 Department of Surgery, Alfred Hospital, Monash University, Victoria, Australia; Nicolaus Copernicus University, POLAND

## Abstract

Capillary flow is known to be non-homogenous between vessels and variable over time, for reasons that are poorly understood. The local properties of individual vessels have been shown to have limited explanatory power in this regard. This exploratory study investigates the association of network-level properties such as vessel depth, branch order, and distance from the feeding arteriole with capillary flow. Detailed network connectivity analysis was undertaken in 3 healthy young subjects using flood-illuminated adaptive optics retinal imaging, with axial depth of vessels determined via optical coherence tomography angiography. Forty-one out of 70 vessels studied were of terminal capillary type, i.e. fed from an arterial junction and drained by a venous junction. Approximately half of vessel junctions were amenable to fitting with a model of relative branch diameters, with only a few adhering to Murray’s Law. A key parameter of the model (the junction exponent) was found to be inversely related to the average velocity (r = -0.59, p = 0.015) and trough velocity (r = -0.67, p = 0.004) in downstream vessels. Aspects of cellular flow, such as the minimum velocity, were also moderately correlated (r = 0.46, p = 0.009) with distance to the upstream feeding arteriole. Overall, this study shows that capillary network topology contributes significantly to the flow variability in retinal capillaries in human eyes. Understanding the heterogeneity in capillary flow is an important first step before pathological flow states can be properly understood. These results show that flow within capillary vessels is not affected by vessel depths but significantly influenced by the upstream feeder distance as well as the downstream vessel junction exponents, but there remains much to be uncovered regarding healthy capillary flow.

## 1. Introduction

The retinal vascular network is made up of a series of vessels of varying size (comprising arterioles, venules, and capillaries) interconnected with each other both laterally and in depth. With the help of these network connections, a physiological equilibrium of blood supply is achieved for the maintenance of healthy retinal tissue. Flow through smaller vessels is particularly important, being the essential site of gas and metabolite exchange, but also because small vessels like capillaries are affected early in the pathology of a spectrum of conditions such as diabetes, hypertension, cardio-vascular diseases, and stroke [1, 2]. For example, capillary changes such as pericyte loss, basement membrane thickening and capillary dropout leading to microaneurysms form the classic signs of diabetic vasculopathy [3, 4]. Spatial and temporal heterogeneity in blood flow is needed to achieve an equilibrium between the local metabolic supply and consumption. The smallest arterioles located close to the capillaries are believed to play a major role in regulating the heterogeneity in capillary perfusion [5]. As described in our earlier study [6], blood flow is highly variable between different capillary vessels and could be influenced by a multitude of factors. Some of these factors can be conceptualized as “local” (within vessel) factors such as the vessel caliber, length, tortuosity, size of the cell-free layer and/or endothelial glycocalyx surrounding the blood column.

In addition to these “local” factors, flow could also be influenced by “network” factors such as vessel arrangement and topography. For example, flow rates generally decrease as blood moves through increasing generations of vessels as major vessels branch into smaller ones (at arteriolar junctions) and as smaller vessels unite into major larger ones (at venular junctions) [7]. For the former, flow velocity is reduced downstream because flow must be conserved across the branch but the overall cross-sectional area downstream is greater. The changes in flow rates brought about occur because flow is conserved at the branch points [8, 9]. This slowing is exacerbated at the microvascular level due to the friction between cells and the vascular wall in single file flow. Dynamic changes also occur down the vascular tree due to variations in pressure as a result of dampening of the pulse wave [7].

In addition to the above, for flow to be efficiently distributed at branch points the downstream vessels should be wide (reducing resistance), however this comes at the cost of a larger metabolic requirement to support the tissue. For a parent vessel of a given diameter, there is therefore some optimal diameter for the daughter vessels to minimize the overall energy expenditure required to maintain a functioning vascular system. Under the assumption of Poiseuille flow expected in large vessels, the optimal diameter is expressed by “Murray’s law” which stipulates that the cube of the parent branch diameter should be equal to the sum of the same quantity for the daughter branches [10, 11]. This is otherwise referred to as the “junction exponent” having a value of 3.0. Murray’s law has indeed been confirmed to hold with reasonable accuracy for larger vessels, i.e. junction exponents at large vascular bifurcations are typically around 3.0 which indicates efficient evolution of the vascular system.

However, in small to medium sized vessels (for example in vessels ranging from >10 to <180 μm diameters) in both human and mouse retina junction exponents less than 3 are typically reported [7, 12–14]. Such departures may be expected in the microvasculature as the effective viscosity of blood is known to decrease for vessels less than ~300 μm in diameter [15], which should favor the adoption of narrower daughter branches at a given bifurcation. As vessels become smaller still, this trend is known to reverse i.e. viscosity increases sharply [16]. Early reports predict this to occur for vessels at around 5–10 μm diameter, whilst more recent reports predict a larger threshold [12, 17]. At present, due to a lack of *in vivo* experimental data it is not known what the junction exponent should be for the retinal capillaries.

Another network factor that might influence flow is the location of a vessel amongst different retinal layers. Capillary densities are known to vary at different retinal depths, with the deep plexus (supplying the retinal layers below inner nuclear until the outer plexiform layer) and intermediate vascular plexus (at the inner plexiform and nuclear layers) having the highest density of capillaries, relative to the superficial vascular plexus (which is located at the ganglion cell layer) [18–20]. The classic trilaminar arrangement detailed by Campbell, Zhang [21]., shows that the retinal vessels have “hammock”-like arrangements in distinct capillary plexi—the superficial, intermediate, and deep capillary plexus are connected with each other through vertically oriented major arteries and veins [21]. However other models suggest that the superficial capillary plexus (SCP) is arranged as a series of hammocks between a major retinal artery and a vein, whereas the deep capillary plexus (DCP) is arranged as a radial capillary converging into a central vortex venule draining into a major venule suggesting that the DCP drains independently into the venules with some connections to the SCP [22, 23]. In contrast, other studies suggest that DCP drains directly into the retinal vein with no obvious interactions with the outflow channels of SCP and ICP, and that only a supply of small arterioles from the ICP nourishes the DCP [24, 25].The dense arrangement of the vascular network in deeper layers may facilitate the metabolic demands of the retinal tissue by increased perfusion. Therefore, it could be the case that flow varies with the axial position (i.e., depth) of a vessel. However, this remains to be established in smaller vessels.

Certain retinal vascular plexi might have increased susceptibility to vascular damage, for example deep capillary network has been implicated as the major locus in the genesis of diabetic microaneurysms and para-central acute middle maculopathy [26–28]. Thus it is of interest to observe the changes in flow parameters as a function of vessel location both laterally and in depth. In another study using optical coherence tomography angiography (OCTA) imaging in retinal vein occlusions show that the deep capillary plexus is more affected (seen as non-perfusion areas) relative to the superficial plexus [29]. These differences in disease presentation could be related to differences in perfusion pressure and the oxygen supply between different vascular layers. The SCP is directly connected to the arterioles, which possess a higher perfusion pressure and oxygen supply [22, 23, 26]. On the other hand, the DCP is considered the primary site of venous outflow, and indeed is known to be affected more in venular occlusions due to drop in perfusion pressures [23, 26, 30].

In summary, there are a number of network-related factors that might influence the flow within a capillary vessel and in a given vascular network. The aim of this study is to consider explanations of the spatial flow variability detailed in our earlier study [6], in terms of the following network variables: vessel depth; branch order; distance from a feeding arteriole (defined as the main feeder of diameter greater than 8μm which supplies the rest of the system); and junction radius (i.e. considering the relative diameter of upstream and downstream vessels at each bifurcation).

## 2. Methods

The study subjects and the imaging procedures used in this study have been explained elsewhere [6] and briefly summarized here: The study was undertaken on three healthy individuals (comprising a male and two females, aged 22–23 years). The participants had no known ocular or systemic conditions, had a clear ocular media and a refractive error less than 4 diopters spherical and 2 diopters astigmatism. Retinal vascular imaging was performed using optical coherence tomography angiography (OCTA) (Spectralis, Heidelberg system, Germany) and adaptive optics (AO) system (flood ophthalmoscopy) in the left eye of all study participants. Eyes were dilated using 0.5% tropicamide (Alcon, Geneva, Switzerland) at least 20 minutes prior to the adaptive optics imaging.

The study obtained approval from the local Human research ethics committee (at The University of Melbourne, ethics approval number 1137234); the study adhered to all the tenets of the Declaration of Helsinki. All study procedures were detailed to the participants and a signed written informed consent was obtained before initiating the study protocol.

### 2.1. AO imaging

The study protocols used for AO imaging have been detailed elsewhere [31–34] and are presented briefly here.

Blood flow imaging was performed using a 2560 x 2160-pixel Andor NEO sCMOS camera (Andor Technology PLC, Belfast, UK), that facilitated image acquisition rates of 200–300 frames per second. The camera’s global shutter mode was used to expose all the pixels simultaneously to minimize distortion due to motion of the eye. A Hartmann-Shack wavefront sensor (Adaptive Optics Associates, Cambridge, MA) was used to measure the ocular wavefront aberrations, and a 97-channel deformable mirror (Alpao, France) was used to correct the measured wavefront slopes in real-time using custom Matlab software. The defocus component (in 0.05D steps) of the adaptive optics correction was adjusted to achieve the best subjective focus for the capillary bed [6].

The imaging was done using different frame rates of 200 or 300 frames per second (fps) for a maximum of 3.4 seconds per acquired video. The number of frames per location were about 1000 (for data imaged at 300 fps) and 600 (for 200 fps data). Retinal locations were chosen close to the edge of the foveal avascular zone, with a preference for locations in which capillary flow could clearly be seen over a large number of vessels across the field (i.e. we biased our choice of tissue away from the larger vessels and away from regions showing great stratification into vascular layers at varying depths).

#### 2.1.1. Imaging procedure

A 750-nm wavelength light was used to image the foveal microvascular network in small regions of diameter 1.25° in a dark room. Fixation was directed around a diffusely back-illuminated black grid on white paper to identify suitable regions of the capillary bed for imaging [6]. The imaging light adhered to the maximum permissible exposure standards for continuous illumination at 750 nm over 3.4 sec [35]. An area of 1–2.5° from fixation was imaged around the foveal avascular zone (FAZ), where stratification of the retinal vascular plexi is limited. Our acquisition was not synchronized to activity of the heart, however the variability due to cardiac contractility was ensured by recording acquisitions over 3 seconds (i.e., around 3 full cardiac cycles).

#### 2.1.2. Measuring cell velocities

Using adaptive optics blood flow imaging videos, the velocities of red blood cells in capillaries over time (given by a “velocity plot”) were calculated using a kymograph approach [6, 36]. Vessels which produced aliasing either on the kymograph or upon manual inspection of videos were excluded from the analysis [37]. Within each retinal field we accepted typically 10–15 capillary vessels for flow analysis. The capillaries were defined by a functional criterion of single-file flow [31] and the vessels were typically less than 8μm in diameter similar to previous definitions [34, 38]. The flow parameters (defined using raw velocity plot and curve-fit measures) were similar to those reported in our earlier study [6]. Among several variables described in that previous work, here we consider those deemed most significant i.e. the maximum velocity (V_max_raw_), minimum velocity (V_min_raw_), average velocity (V_ave_) and pulsatility ((V_max_ ‐ V_min_) / (V_max_ + V_min_)) [6]. *“The variability in velocities between vessels across the network is referred to as ‘spatial heterogeneity’; this was studied by examining the capillary vessel flow patterns from neighboring retinal fields surrounding the FAZ*” as given in our previous study [6].

#### 2.1.3. Vessel diameters

The vessel diameters were measured using motion contrast data together with an automatic thresholding approach as described recently ([6]). “Division” images were computed to identify the extent of the internal vascular lumen [39]. This allowed vessel centerlines to be traced manually, with the extent of the lumen quantified using an automatic thresholding method applied to a small region of interest surrounding each vessel [40]. Thresholded pixels were expressed by their distance from the vessel centerline, with a maximum likelihood curve fit providing an estimate of overall lumen diameter.

To capture the variability implicit in the quantification of vessel diameter due to sources of error such as noise or blur, we developed confidence limits by bootstrapping the straightened “division” image for each vessel. On each iteration of the bootstrap, rows in this image (i.e. different cross-sections through the vessel) were randomly drawn with replacement and the vessel diameter re-computed. The mean vessel diameters noted were 4.33 ± 0.9 μm [6], the average and standard deviation for the 95% lower and upper confidence limits were 3.91 ± 0.72 and 4.74 ± 1.12 respectively.

### 2.2. Vessel depths

The vessel depths for each vessel of interest (VOI) were determined using OCTA (Spectralis, Heidelberg Engineering system, Germany) and the accompanying Heidelberg Eye Explorer software.

**Fig 1:**
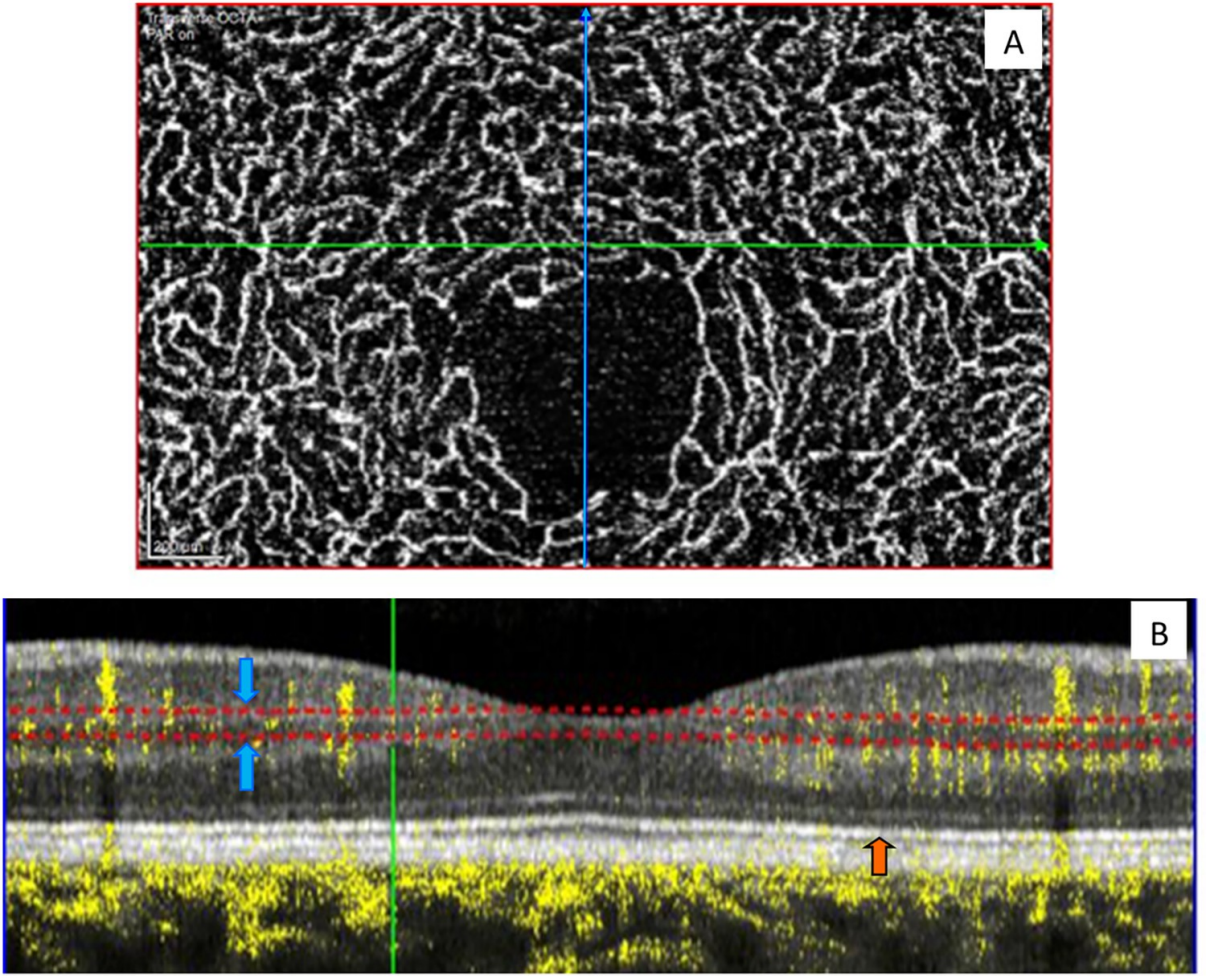
OCTA extraction for vessel depth analysis. **A**: Enface image of the OCTA data showing perfused vessels of the chosen slab thickness. Crosshairs are positioned at a vessel segment of interest located 1.25° superior to FAZ (green line represents transverse scan and blue is the orthogonal scan). **B**: A close up view of Structural OCT section images with overlay of OCTA data in yellow, showing thickness slab (in red dashed lines pointed by blue arrow) positioned 160 microns away from photoreceptor layer 1 (indicated by orange arrow).

For analyzing vessel depths, the contrast setting in the software was set to 1:2 and the software’s projection artifact removal feature was used to minimize any artifactual flow signals resulting for deeper vessels. The retinal tissue was scanned in depth using slabs of 40 μm thickness spaced 10 μm apart. This slab thickness was found empirically to optimize capillary localization in depth, trading off signal-to-noise ratio with axial precision. Depth was computed using photoreceptor layer 1 (PR1) as a reference, which corresponds to the hyper-reflective line representing the boundary between the photoreceptor inner and outer segments [41]. An example is shown in Fig 1 depicting the position of a 40μm slab (shown in red) located 160 microns above the PR1 layer on an OCT cross-sectional image (Fig 1).

**Fig 2:**
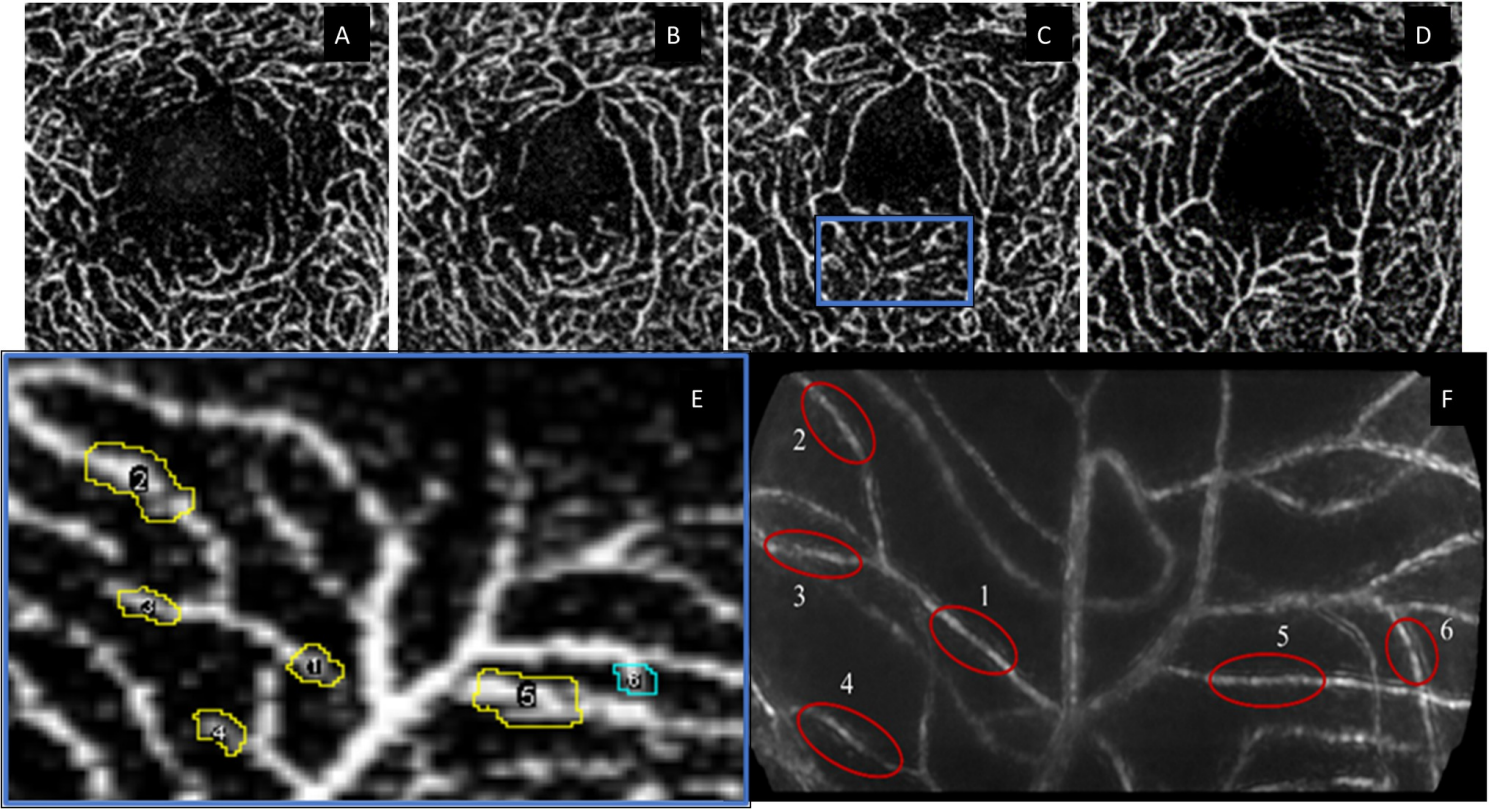
Evolution of vessel intensity over OCTA slabs located at different depths and vessel drawings. **A**: 130 (depicting deep vascular plexus), **B**: 150, **C**: 170 and **D**: 190 microns away from PR 1 layer (depicting superficial vascular plexus), **E**: An inset from C (in blue), drawing vessel ROI markings for pixel intensity analysis on an OCTA map. Different VOI indicated by the numerical code at the center of the marking. **F**: Reference AO DIV image with vessels labelled numerically that matches with E.

The depth of vessel segments were automatically quantified using Image J software [42]. For each subject, 40 μm slabs extracted from OCTA were imported as stacks in Image J. Slabs were positioned spanning a range from 60 μm away from the PR1 layer (defined by the outer edge) to 220 μm away from this layer, in 20 μm steps. All vessels studied were observed to emerge and then fade out within this depth range. Some examples of these slabs showing the evolution of pixel intensity at different depths are shown in Fig 2A–2D.

**Fig 3:**
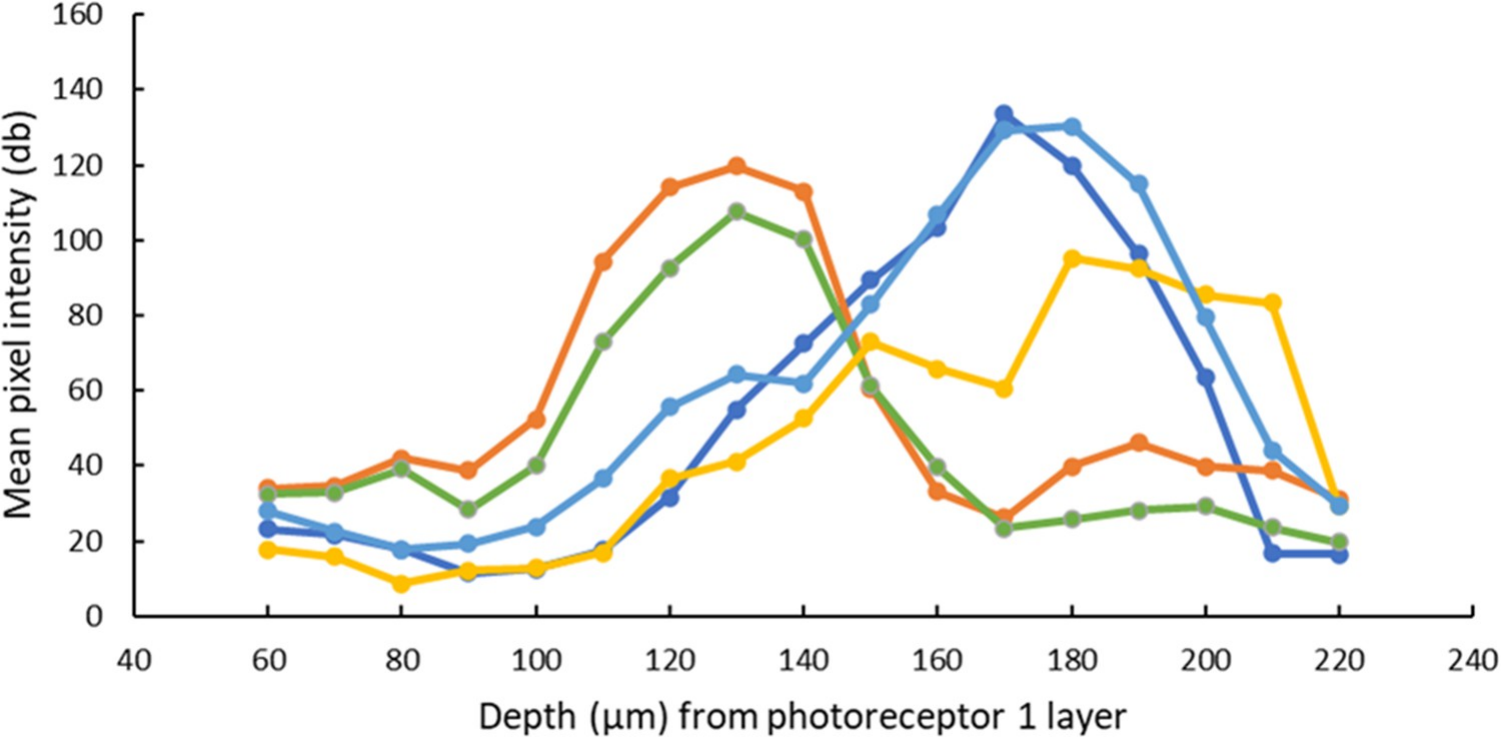
Variations in pixel intensity of vessel segments with differing depths. Data from subject 1 showing depth and pixel intensity information for vessels segments within a retinal field 1.75° inferior & 0.75° temporal to FAZ. Different vessels are indicated by unique colored lines.

To quantify vessel depth, each VOI was identified using the division (DIV) image as a reference (Fig 2E and 2F). Once the VOI was clearly visible on one of the stack positions, the vessel outline was marked using a free hand tool in Image J (e.g. Fig 2E). A few measurements were set for analysis within the software: these include the area, mean gray value, minimum and maximum gray values of the pixels marked and the standard deviations. The stack position that showed highest mean gray pixel intensity was observed as the depth (in μm) of a vessel segment, and was identified by plotting the changes in mean pixel intensity as a function of the retinal depth (shown in Fig 3; the intensity of a given vessel is seen to follow a bell-shaped curve with peak intensity at a particular depth and diminishing vessel intensity on either end).

### 2.3. Network mapping analysis

Once the depth of all vessels of interest was determined as explained above, the relation of each VOI with that of the feeding arteriole in a network was established. This included the calculation of the total distance along the flow axis, and the number of branches to determine the branch order, for each VOI relative to its feeding arteriole.

**Fig 4:**
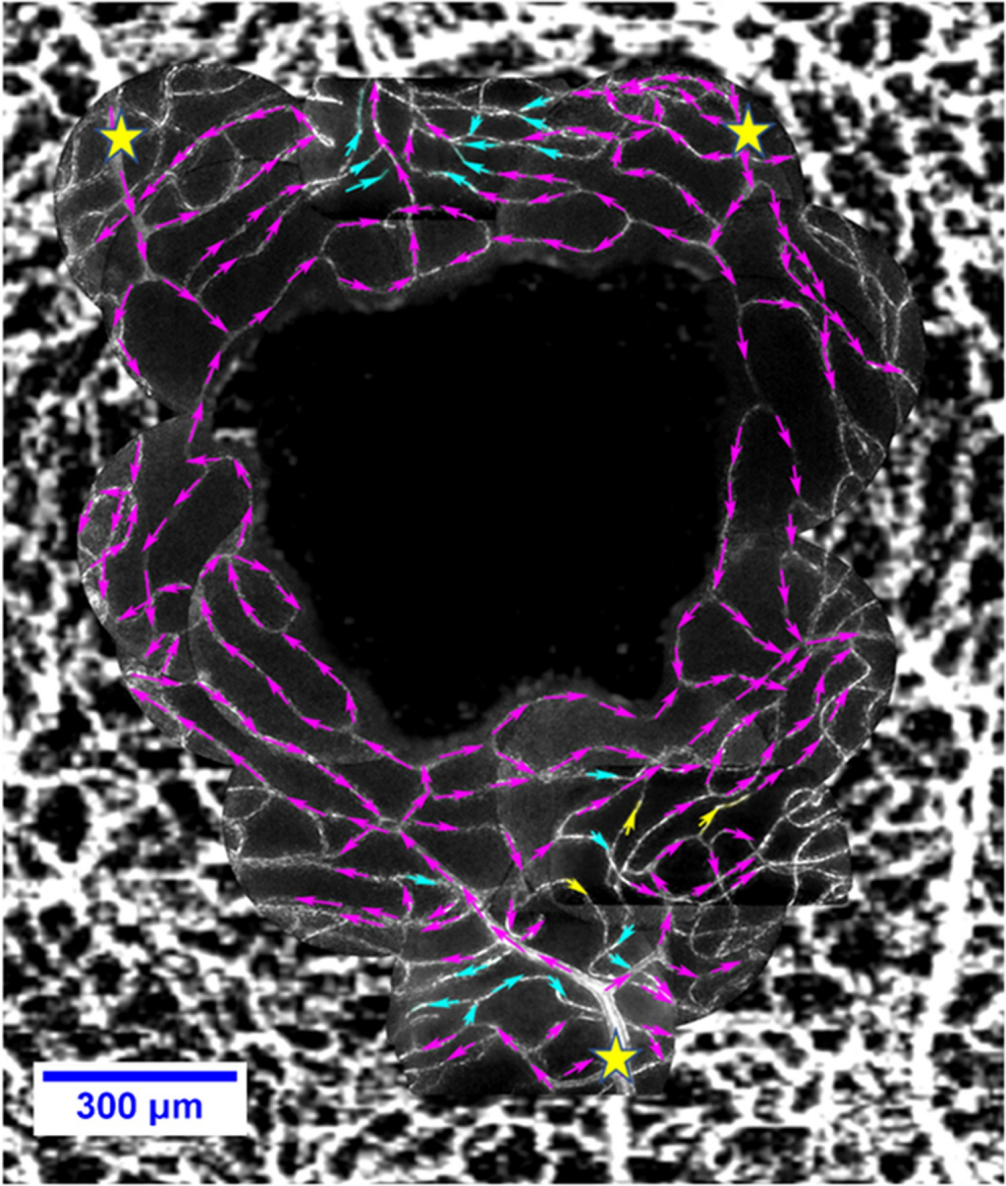
Establishing vessel connections. AO montage overlaid on and registered with OCTA data for Subject 1, arrows indicate blood flow direction, arrows in cyan and yellow indicate vessels of interest, cyan represent deeper vessels, yellow represent superficial vessels, magenta indicates unknown depth & non-VOI, yellow asterisks represent feeding arterioles.

To determine these factors, a detailed analysis of the network topography was performed using AO montages for each subject surrounding the foveal avascular zone (FAZ). Firstly, all the vessel connections with each other were established by carefully examining the blood flow through these vessels using the high speed adaptive optics video data together with the animation and frame scrubbing features in Image J. By observing the direction of flow from one vessel point to the other separated by branch points and at crossings, vessels were labelled as “parent” (blood flowing from) or “daughter” (blood flowing to) of a reference vessel. Flow direction and connectivity were only recorded where they could be confidently established, for example without evidence for corruption by aliasing. Estimation of flow is more vulnerable to aliasing where cells are evenly sized and spaced; our lengthy data acquisition (~3 sec) ensured that regular flow was often punctuated with longer stretches of plasma and/or erythrocyte aggregates and/or leukocytes which helped to guard against the effects of aliasing. The long acquisition also ensured that flow could be observed during the diastolic portion of the cardiac cycle where flow is slowest and so the direction of flow for a given vessel was easiest to confirm without aliasing. Fig 4 shows one AO montage overlaid on and registered with OCTA data from the same area. The direction of movement of blood through different vessels in the network (purple) and the location of VOI in superficial (yellow) and deep (cyan) capillary plexi are indicated in Fig 4.

As explained above, the connections for all individual vessel segments in a network were established as a parent or a daughter to every other vessel. The vessel traces were drawn out for all the segments using a single pixel pencil tool in Adobe Photoshop. A minimum of 1-pixel gap between junctions and crossings was created while drawing traces to allow standard image processing tools to easily delineate vessel segments.

**Fig 5:**
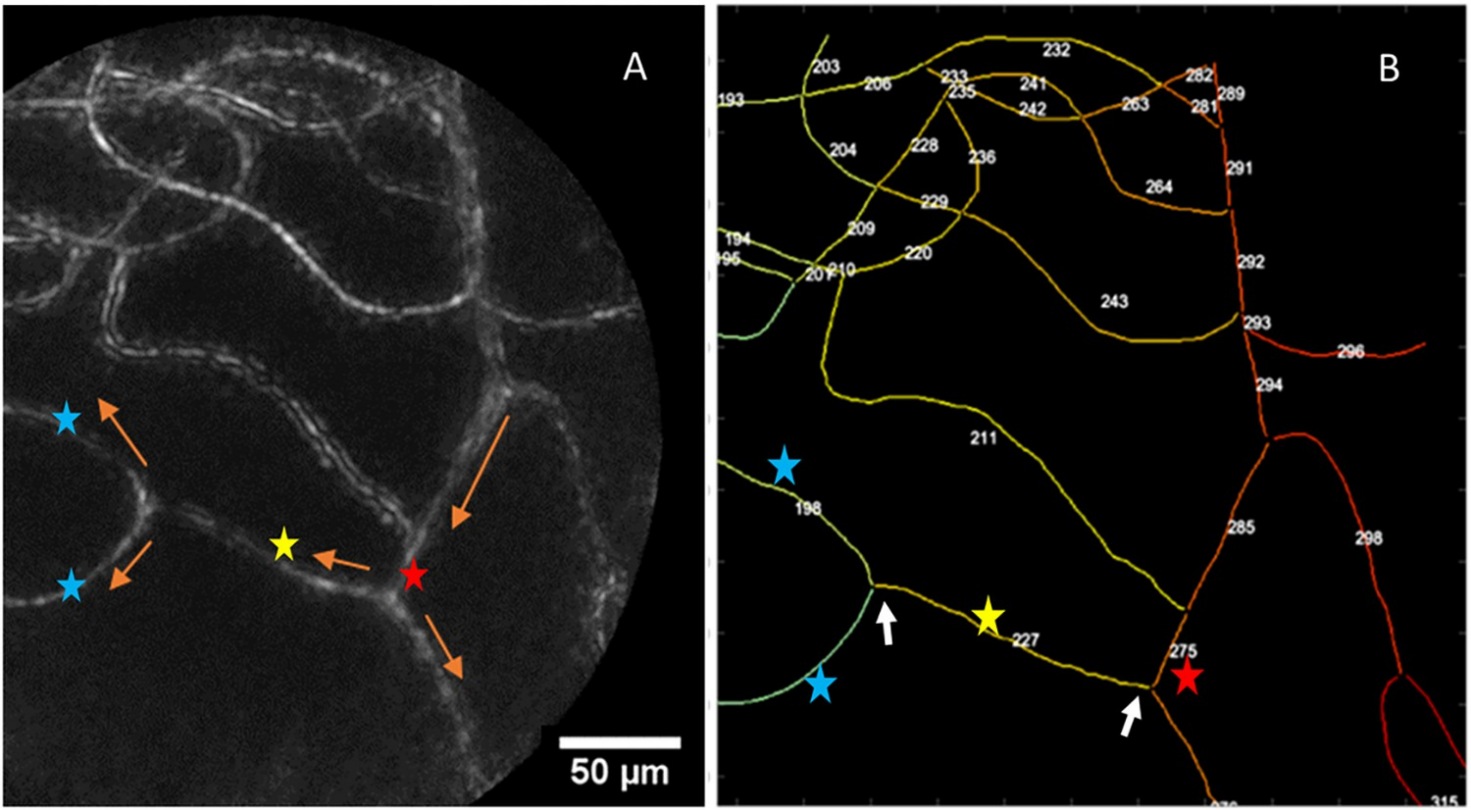
Vessel identifiers used to map network connections. **A**: Showing motion contrast image of a supero-temporal field from subject 1. Showing the relationship of VOI with the upstream (parent) vessels and the downstream (daughter) vessels. The radii of these vessels were used to calculate the Junction exponents. Arrows indicate flow direction. Vessels of interest indicated by yellow star, parent vessel in red star, and daughter vessels represented by blue star. **B**: The numbers and the color code indicate unique ID for each vessel segment (reference to the DIV image in A) separated by a branch (indicated by white arrows) or a junction/crossing.

The binary traces from the network were processed using the “bwlabel” tool in the Image Processing Toolbox of MATLAB R2017a (The Mathworks, Natick, USA). This provided a unique numerical label for all traced vessels, as shown in Fig 5. These ID’s were stored in a spreadsheet database and were also used to map out the parent-daughter connections.

We recorded the following parameters for each vessel in a spreadsheet for analysis:

1. Distance of the vessel from the feeding arteriole (that is, the length of the path taken to reach the vessel, potentially through multiple branches, from its feeding arteriole).
  The distance of the VOI from a feeding arteriole was defined as the distance between the upstream of the feeding arteriole to the center of the VOI, calculated by adding the individual vessel lengths.
2. Branch orders: The branch order was calculated as the number of branching points a given vessel of interest is located away from the feeding arteriole, where the feeder arteriole itself is considered of order “1” and the order number increases for each vessel separated by a branching point. With 1 being the highest order, in this data the most remote branch was of order “9”.
3. Classifying the vessel segments—Capillaries were classified based on their connections to arteriolar/venular junctions (detailed in section 2.4).
4. Radii of the vessels at each bifurcation junction to fit the junction exponent for evaluation of Murray’s law.

Further information on the last two points is given below.

### 2.4. Classifying vessel segments

**Fig 6:**
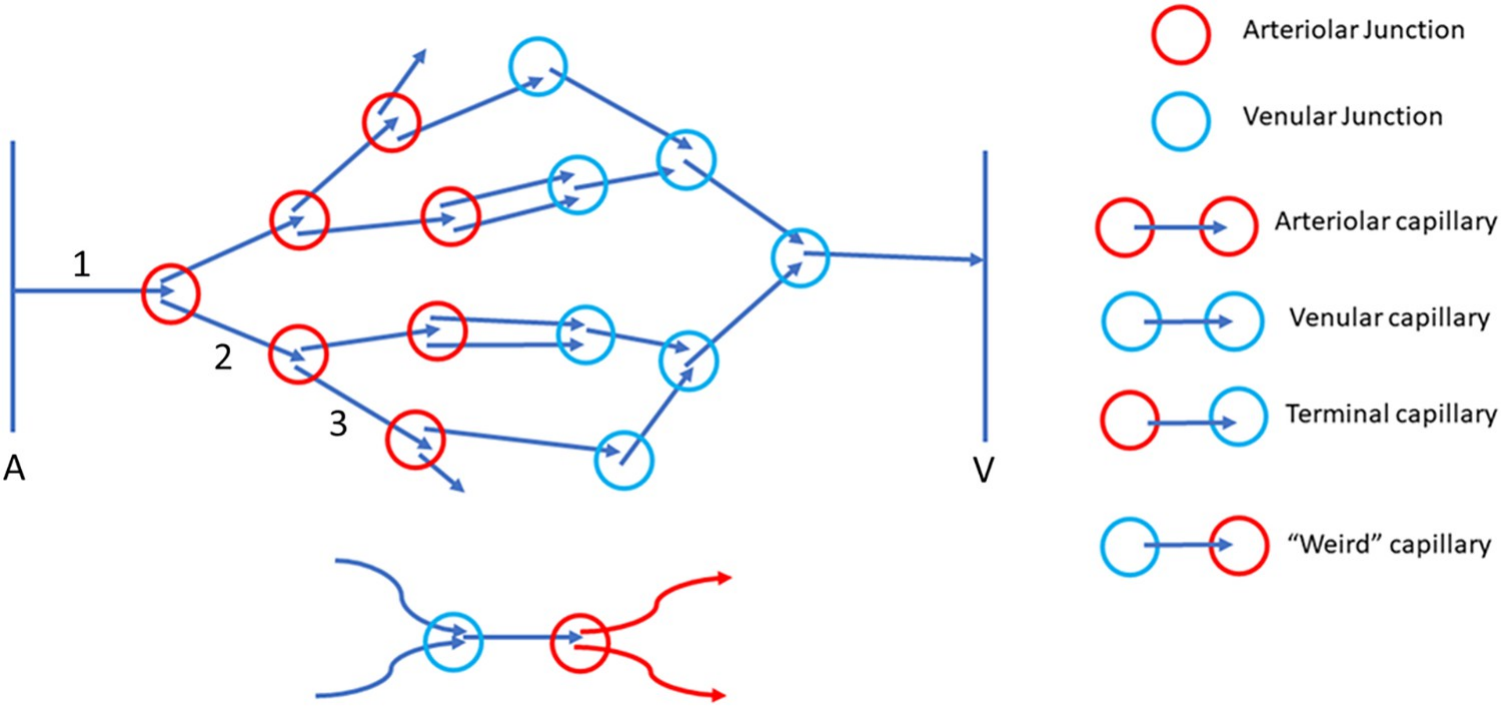
Classification of capillary types. An arteriolar junction is defined as a point where a supplying vessel branches out to two daughter vessels, and a venular junction is defined as a point where two minor vessels drain/unite into a collecting vessel. The numbers indicate branch orders, A–Arteriole (feeder), V–Venule (collector).

The vessel segments were classified by way of their connections with each other within a capillary network (Fig 6). These connections are described as follows:

1. Arteriolar capillary (AC): A vessel segment arising from a single parent vessel and giving rise to two daughter vessels. It can also be defined as a capillary segment between two feeding/arteriolar junctions.
2. Venular capillary (VC)–A vessel which has two parents and a single daughter vessel. It can also be defined as capillary between two collecting junctions.
3. Terminal capillary (TC)–A vessel segment connecting an arteriolar and a venular capillary, that is which has one arteriolar capillary as a parent and a venular capillary as a daughter vessel.
4. “Unorthodox” capillary (WC)–This is similar to a terminal capillary, however the venular junction appeared at the start of the vessel and the arteriolar junction at the end which is not commonly present in a vascular network and hence termed “Unorthodox”.

### 2.5. Fitting junction exponents to study Murray’s law

Murray’s law predicts the diameter of parent and daughter vessels at each vascular junction in order to minimize the total energy required to support propagation of the blood column (wider vessels preferred) and nourishing the vascular tissue (thinner vessels preferred). The law states that the sum of the daughter vessel branch radii should be related to the parent vessel branch radius by an exponent of 3.


$$R_{parent}{}^{3}=R_{daughter1}{}^{3}+R_{daughter2}{}^{3}$$ (1)

Where R_parent_ is radius of the parent vessel, R_daughter_ stands for radius of the daughter vessels 1 and 2. Parent vessel at a supplying junction indicates the major vessel which supplies blood to two minor daughter vessels, whereas ‘parent’ at a collecting junction indicates the major vessel, which receives blood from the two minor daughter vessels.

Junction exponents were calculated using the radii measurements of the vessels at the junctions connected to each VOI. Fig 5 shows the identification of vessels at a junction in relation to the VOI which includes the parent and daughter vessels. The radii of these vessels were measured so that junction exponents could be determined as explained below:

First, a check was made to verify that the vessel which should classically be the widest (upstream at arteriolar junction; downstream at venular junction) was in fact the widest. Murray’s law as usually defined is not able to support a major vessel which is narrower than the other vessels.

Next, we trialled different junction exponents replacing the cubic operations in Eq (1) to determine the best fit to the data. For example, in the following Eq (2), we have evaluated ‘x’ (the junction exponent) on the range 0 to 10. This was done to determine which exponent best predicted the radii of the parent vessel from the two daughter vessels.


$$R_{parent}{}^{x}=R_{daughter1}{}^{x}+R_{daughter2}{}^{x}$$ (2)

The exponent minimizing the sum of squared errors (SSE) was chosen:

$$SSE=\sum_{x=0}^{10}{\left(R_{parent}-{\left({R_{daughter1}}^{x}+{R_{daughter2}}^{x}\right)}^{\frac{1}{x}}\right)}^{2}$$ (3)

Brute force optimization was carried out to determine the junction exponents. We trialled candidate values for the junction exponent to find the value which best matches the observed vessel radii. We choose the best fitting one as defined by minimum error.

The goal of this model-fitting operation was that for each trialled junction exponent we used the daughter radii to predict what the parent vessel radius should be. Then, we compared this to the actual measured radius. The junction exponent giving the minimum error relative to the predicted value was selected.

Where the junction vessel radii were available to fit the junction exponents (JE), the vessels are described below as “JE_fit”. For vessels where fitting the exponents was not possible due to non-availability of one of the vessel radii, or where the parent vessel was not the thickest, they are categorized below as “JE_non-fit”. Junction exponents were fit to both the supplying (JE_upstream) and the collecting (JE_downstream) ends where applicable, which across all junctions analyzed always corresponded to arteriolar and venular junctions respectively.

### 2.6. Statistical analysis of the data

The statistical analysis of the data is similar as that explained in our previous study [6]. We have studied the distribution and associations of network variables such as vessel depths, branch orders, feeding arteriolar distance, junction exponents with the flow parameters. Statistical analysis of the data was performed using IBM SPSS statistics for windows, version 21.0 (IBM Corp., Armonk, N.Y., USA). Correlations between parameters were examined using the two-tailed Pearson’s test at a significance level of 0.05. Correlation coefficients between values 0.00 to 0.30 –were considered as negligible correlation, 0.30 to 0.50 as low correlation, 0.50 to 0.70 as moderate, and 0.70 to 0.90 as high correlation [6, 43].

## 3. Results

The distribution of measured parameters for the measured variables and an overview of their correlations with the flow parameters is summarized in Table 1, and described in further detail below.

**Table 1:** Illustrating the distribution of network variables and their correlations with the raw flow parameters.

*Network variables*	*N*	*Average*	*Ranges (min—max)*	*Correlations*, *r*			
				*V*_ave_	*V*_min_	*V*_max_	*Pulsatility*
*Feeding arteriole distance (μm)*	31	447	178–817	0.25	**0.46** **	0.14	**-0.43** *
*Vessel depths (μm)*	52	167	130–220	0.27	0.16	0.24	0.17
*Branch orders (n)*	35	6 (median)	1–9	0.04	0.13	-0.05	-0.17
*JE_upstream*	17	2.13	0.85–3.60	-0.26	-0.06	-0.25	0.13
*JE_downstream*	16	2.19	0.75–5.84	**-0.59** *	**-0.67** **	-0.38	-0.1

In bold–statistically significant correlations, *—p<0.05, **—p<0.01, Pearson’s correlation, N–sample size (number of capillary vessels), min–minimum, max–maximum, Vave–average velocity, Vmax–maximum velocity, V_min_−minimum velocity, pulsatility was given by (V_max_-V_min_)/(V_max_+V_min_), JE–Junction exponent.

### 3.1. Vessel depths

**Fig 7:**
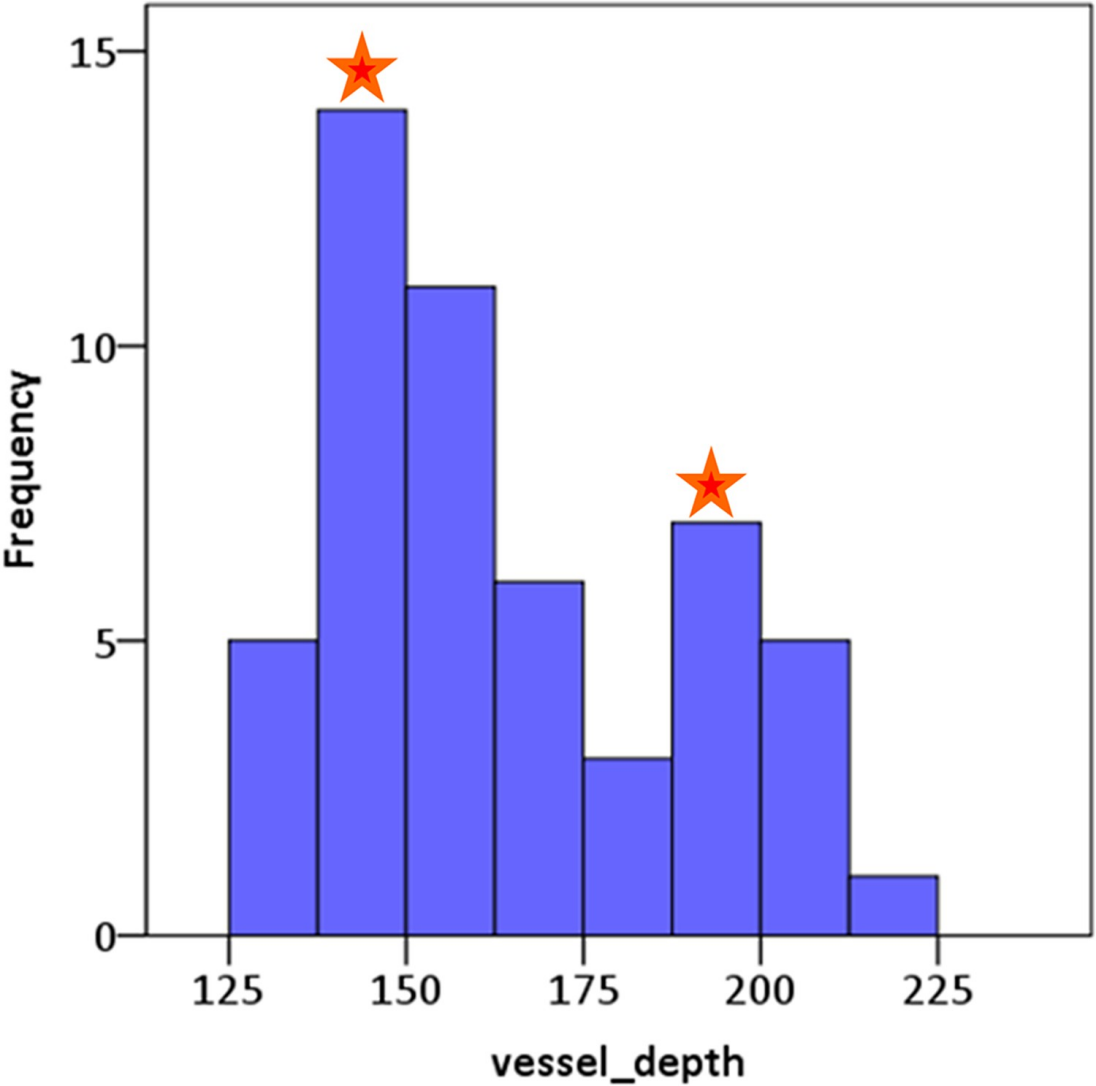
Distribution of vessel depths from all vessels segments analysed. The histogram plot reveals that vessel depths show a bimodal distribution with 2 peaks (indicated by red stars), 1^st^ peak (with a frequency of about n = 14) noted at the deeper layer (at 150 μm away from PR1 layer) and the other peak (a frequency close to 7) at the superficial layer (i.e., at 200 μm away from PR1 layer). The bimodal distribution of vessel depths was also found in a given field (as given in Fig 3).

The vessel depths relative to PR1 ranged from 125 μm– 225 μm across 52 vessel segments from 3 subjects. Fig 7 shows that most vessel segments studied (n = 35, 67%) were located between 125–175 μm away from the photoreceptor layer 1; and 33% of the vessels (n = 17) were located between 175 and 225 μm. It should be noted that further away from the edge of the foveal avascular zone, complete separation into the well-known trilaminar structure of the inner retinal vascular network would be expected, with 3D visualizations of diving vessels. However, there are not many such diving vessels at the area of our imaging as it is quite close to FAZ.

**Fig 8:**
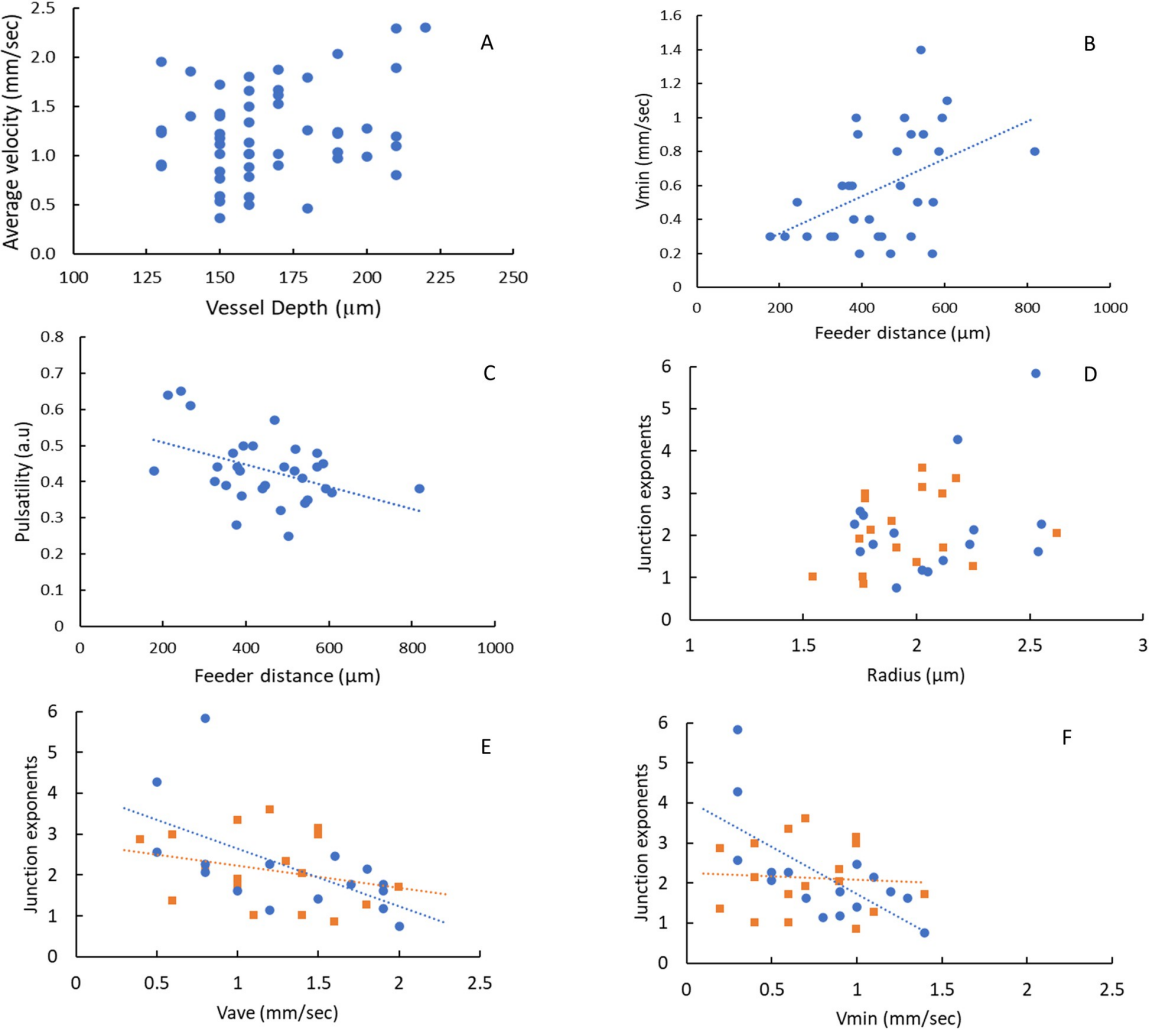
Scatter plots representing some of the relationships between measured variables. **A** shows there was no association between vessel depth and average velocity. **B** and **C** show correlations between flow parameters and distance to the feeding arteriole with an increase in V_min_ (B) and a decrease in pulsatility (C) with increasing feeder distance. **D** shows that junction exponent was not related to vessel radius, whilst **E** and **F** show a moderate correlation between junction exponent and velocity measures (data from upstream vessels represented in orange, downstream vessels in blue).

An example of the non-significant relation between velocity measures and vessel depth is plotted in Fig 8A. Similarly, no significant associations between flow parameters and the branch order were found as given in Table 1.

### 3.2. Vessel junction exponents

Junction exponents were fit for both upstream and downstream junctions for each VOI. Out of 70 vessels, there were 33 vessels for which junction exponents could be fit (JE_fit vessels). Of these, 17 were upstream and 16 were downstream; as mentioned above these corresponded to arteriolar and venular junctions. Over both categories, the JE ranged from 0.75 to 5.84 as shown in Fig 8D, with no discernible difference in distribution between categories.

There was no significant correlation between the JE and the radius of the vessel of interest (which could be either a parent/daughter vessel) (r = 0.24, p = 0.36 for upstream and r = 0.35, p = 0.18 for downstream vessels, Pearson’s correlation) (Fig 8D). Very few vessels were observed to match Murray’s law i.e. to have JE near 3, with most vessels having a much lower exponent (Fig 8D).

Of the flow parameters, a moderate negative correlation of JE with velocity measures was found (V_ave_: Pearson’s r = -0.59, p = 0.015; V_min_: r = -0.67, p = 0.004) for the downstream (venular side) junction, whereas upstream junctions showed a poor correlation of JE with the variables (V_ave_: r = -0.26, p = 0.29; V_min_: r = -0.06, p = 0.80) (Fig 8E and 8F).

Since the correlations between JE and velocity measures for the downstream vessels (noted in Fig 8E and 8F) may be influenced by two outliers (e.g. JE above 4), the analysis was repeated after removing these two outliers from the downstream vessels. It was reassuring to see that the correlations between JE and V_min_ still and JE and V_ave_ remained statistically significant with correlations of r = -0.57, p = 0.03 and r = -0.52, p = 0.05 respectively.

### 3.3. Classification of capillary types

The relations with the network variables were able to be established for 70 out of 72 VOI from 3 subjects. These 70 vessels were classified as arteriolar/venular/terminal capillaries based on the type of junctions they were emanating from. Out of 70 vessels, 58.6% (n = 41) were terminal capillaries, 5.7% (n = 4) were arteriolar capillaries, 4.3% (n = 3) were venular capillaries. The definitions for these capillary types have been described in section 5.2.3. There were 31.4% (n = 22) vessel that could not be classified into any of the groups as complete information was not available for them. “Unorthodox” capillaries (n = 2) were noted in two of the subjects (one in each).

## 4. Discussion

From our previous study [6], it was observed that the local structural and flow related aspects of the vessel were generally poor determinants of spatial flow variability. In this study the potential influence of more global “network” variables such as vessel depth, remoteness of a capillary segment from its “feeding arteriole” (both in absolute distance and in terms number of intervening bifurcations), and the effect of the relative diameter of vessels at branch points were explored. The influence of each of these variables on the flow are discussed below:

### 4.1. Vessel depths

As explained in the introduction, there appears to be a link between the DCP and venous drainage [23]; which could mean that deeper vessels have slower flow compared to the SCP which would feature a more arterial focus. The muted pulse pressure wave could contribute to the slower flow in the venous system. Due to this reason, we postulated that vessel depth might have associations with the flow parameters, with the capillaries from superficial plexus showing greater pulsatility and flow relative to the capillaries from deeper layers.

However, for our subjects, no significant associations were found between the depth and flow parameters (such as average, minimum, maximum velocities and pulsatility). There might be a few reasons for this: firstly, our dataset does not feature a wide range of vessel depths as the vessels studied are mostly located in the intermediate to deeper layers, with a few in the SCP (in addition the vessels are located close to the FAZ where there is not much stratification of the plexi). As explained above, vessels that support faster blood flow are more likely to be found in the SCP. In this respect, the data set presented in this study is limited; a larger range of vessel depths might reveal an association between flow and vessel depth, but such an association does not emerge from the evidence presented here.

### 4.2. Feeding arteriole distance

The anatomical arrangements of the capillaries in relation to the parent (or feeder) arteriole might also influence flow due to variations in path length together with the accrued loss of energy in deforming cells and/or the vessel wall. The vessel wall moves due to pulse wave propagation, hence, pulse energy is lost in deforming the vessel wall, as well as there is energy lost due to friction of movement of flow through the vessel. This energy loss is expected to accrue the longer the length of the vessel. Flow differences between neighboring vessel segments (as noted in our previous study [6] might then arise due to each segment’s proximity to its feeding arteriole. This was confirmed in our data where we observed that in general, as cells travel a greater distance along the network, the flow pulsatility is lowered which suggests a progressive dampening of the cardiac imprint along the vascular tree over typical distances of a few hundred microns. This is similar to the study by Joseph et al. [7]., who also used adaptive optics technology to study blood flow dynamics in the living mouse retina and observed that the flow varied along the progression of vascular tree with decreasing flow rates seen in increasing vessel orders. They demonstrated that pulsatile flow varies for vessel types and locations and that even in same generation vessels, venules demonstrated a lower pulsatility than arterioles.

Significant dissipation of pulse wave energy in the microvasculature has been postulated previously due to the much slower pulse wave velocities in the microvasculature as compared with established values in larger vessels of the body [44]. The measures of normal pulsatility, established here in healthy individuals, will help to set a baseline for future studies of vascular disease, where stiffening of the vessel wall may manifest in less energy dissipation and hence in greater pulsatility (as well as pulse wave velocity).

Reductions in pulsatility with feeding arteriole distance were accompanied by a rise in V_min_ as might be expected, however we did not see a commensurate drop in V_max_. This indicates that dissipation of pulse energy may be asymmetric between the rapid pressure spikes of the systolic phase and the slower relaxation of the vessel during the diastolic phase.

### 4.3. Capillary type

Each parafoveal microvascular network in this study was comprised of a combination of arteriolar capillaries, venular capillaries and interconnecting terminal capillaries. “Unorthodox” anastomoses were occasionally noted (in 2 subjects) which indicates collection followed by subsequent re-distribution. However, the majority (58.6%) of vessels studied here were of terminal capillary type i.e. with collecting and supplying junction vessels on either end. This does not indicate that most capillaries in the retina are of this type, however these formed the majority of vessel segments that were amenable to the velocity analysis described in this study.

### 4.4. Junction analysis and Murray’s law

Some junctions could not be fit with Murray’s law (52.85%, 37 out of 70 vessels) because the parent vessel of that junction appeared narrower than the daughter vessels. This could indicate that the vessels deviated dramatically from Murray’s Law-like behavior where the main/parent vessel is actually the widest in diameter; however, the result could be obtained in some cases due to imaging artifacts altering the apparent width of vessels.

Studies show evidence that vessels of diameters ranging 20–100 μm can deviate from the cubic relationship shown in Murray’s law; studies have shown an exponent of two or less (ranging 1.58 to 2.94) in healthy vessels as imaged by AO [12]. In such larger vessels, deviations from the expected cubic relations between parent and daughter vessel radii may arise due to significant changes in blood viscosity or vessel stiffness, and in disease conditions where there is a flow redistribution across the network [11, 12]. From the current results, the junction exponent was also typically around 2 in vessels of diameters less than 10 μm.

Murray’s law is predicated on the principle of energy minimization; the higher the vessel diameter, the lower the energy needed to drive the flow but the higher the energy needed to maintain the fluid volume and associated vascular tissue [10]. Smaller junction exponents have been reported for retinal vessels of diameters ranging from 10 to 180 μm [7, 12, 13]. This has been proposed to occur due to the Fahraeus-Lindquist phenomenon whereby the effective viscosity of blood in vessels narrower than around 300 μm diameter (i.e. all retinal vessels) is reduced compared to that seen in larger vessels [12, 15]. This means that vascular resistance is proportionally less at smaller vascular bifurcations, favoring narrower daughter branches in order to minimize the total energy spent supporting a functioning vascular system (as described in the Introduction). For even smaller vessels like capillaries there is less energy needed for maintenance of blood, however their high resistance means that more energy is required to propel the blood forwards. This is compounded by strong cell-wall interactions in the capillaries, which should elevate resistance beyond what is implied under Murray’s law (which presumes Poiseuille flow). Hence at any single junction, one might expect vascular branches to be proportionally wider (increased junction exponent) at capillary bifurcations compared with exponents previously reported for larger vessels. In fact, at bifurcations for which an exponent could be fit our results show junction exponents comparable to the values reported above for larger retinal vessels.

Within the framework of Murray’s law, where total energy to support the vascular system is minimized, an increase in energy required for blood flow (due to narrow daughter branches) should be balanced by a reduction in the total energy to nourish the vascular tissue. This may come about due to the very high spatial density of capillaries in the microvasculature, where the total cross-sectional area is much higher than elsewhere in the vascular tree [9]. Therefore, one reason for the evolution of such narrow capillary branches, despite the increased energy cost of pumping blood through them, may be to reduce the energy cost required to support the vascular infrastructure. Minimization of energy expenditure in this way may be further supported by the generally smaller wall-to-lumen ratio of the capillaries [45, 46], which implies less vessel tissue per volume of blood and therefore lesser need to nourish the vessel [11].

Beyond the framework of Murray’s law, it is important to note that the exchange of metabolites between blood and tissue occurs predominantly in the narrowest vessels. This is not a factor for the large vessels where Murray’s law is known to hold (i.e. a junction exponent of 3.0). Thus, there is an extra consideration in the optimal “design” of the vascular tree, which is that it may be considered worthwhile to spend extra energy on supporting narrow daughter branches (e.g a junction exponent of 2.0) due to the commensurate increase in diffusion efficiency which is, after all, the end goal of the entire cardiovascular system.

In vessels that were able to be fit with Murray’s law, we observed a moderate inverse correlation between flow velocity measures and the junction exponent at venous bifurcation. This means that flow was faster where two capillaries fed into a significantly wider collector, and conversely that flow was slowed where two capillaries fed into a collector of similar diameter. The same associations were not significant for the arteriolar junctions, which could suggest that flow velocity for terminal capillaries tends to be limited by outflow considerations.

### 4.5. Study limitations

Following the framework provided by predictions according to Murray’s Law, calculation of junction exponents require that parent and daughter vessels are determined very accurately. It is necessary that there is enough resolution such that accurate diameter measures can be made, in order to claim anything about the validity of parent-daughter size relationships based on Murray’s law in this experimental paradigm. Fundamental constraints on vessel diameter measurements are imposed by the spatial resolution of the imaging system in combination with data analysis approaches used, as described below. The spatial resolution of the device is approximately 2 μm, however diameter measures consider a series of points along each vessel which can be expected to improve precision. For example, a 6 μm vessel readily appears wider than a 5 μm vessel even though the difference in vessel diameter is less than the spatial resolution of the instrument.

A generalized boot-strapping method performed in Matlab was used to return confidence intervals for our diameter measures (as described in Methods). These confidence limits were typically within 2.9 to 10.4 μm, giving us some assurance that valid conclusions can be drawn regarding the apparent junction exponent and hence the applicability of Murray’s law at the capillary level.

The mean ± SD diameter measurements of our data as reported in our previous study was about 4.33 ± 0.90 [6], this is comparable to previous findings in capillaries which show a mean ± SD diameter of 5.5 ± 1.3 [34]. In addition, we note that the diameter measurements may seem smaller than expected as the values reported solely include the lumen, specifically the cell-containing part of the blood column and not the vessel wall (which may attribute to approximately 2 microns of additional thickness including the plasma layer and the endothelial cell layer).

## 5. Conclusions

Previous studies have explored the influence of local capillary parameters, such as lumen diameter, on microvascular flow. Here, we explored the influence of network-level factors including retinal strata, vascular branching order, distance from the feeding arteriole, and the relationship between parent and daughter diameter at bifurcations (Murray’s law). The vascular supply must meet the unique physiological demands of each of the retina’s lamina through a brief, temporally integrated period. Although it would be expected that the farther away deeper capillaries have slower flow, and possibly reduced oxygenation, expectations are not always met, as our results show no significant association of flow parameters with vessel depth. The capillary vessel pulsatility was reduced with increasing distance from the feeding arteriole, correlated with an increase in diastolic velocity. This may be indicative of the dissipation of pulse energy along the microvascular tree. At many bifurcations the “daughter” vessels did not appear to be narrower than the parent, suggesting stark departure from Murray’s law and consistent with high resistance in narrow vessels. For bifurcations that could be fit with Murray’s law, junction exponents averaged approximately 2.2. This is comparable to recent reports for arterioles in the range 20–50 μm but larger than that reported for venules in the same size range, indicating that the capillaries are relatively better matched in diameter to their collecting venule. We also observed a negative correlation between junction exponent and flow velocity at collecting bifurcations, indicating that faster capillary outflow is permitted by correspondingly wider venules. There was no such association at the upstream, arteriolar, end which suggests that outflow resistance may generally be more important in determining flow velocity for the capillaries. These observations will guide the future development of flow models to predict microvascular network flow dynamics in health and disease.

## Supporting information

S1 File Data showing network analysis. (XLSX)
